# Host Cell Protein MCM7 Interacts with NP1 of Minute Virus of Canines and Facilitates Viral DNA Replication

**DOI:** 10.3390/microorganisms13092154

**Published:** 2025-09-16

**Authors:** Zhiping Hei, Xiang Ren, Kai Ji, Zhijie Zhang, Binghan Chen, Yuning Sun

**Affiliations:** 1 Department of Biochemistry and Molecular Biology, School of Basic Medical Science, Ningxia Medical University, Yinchuan 750004, China; hax163guoker@163.com (Z.H.); 13895482903@163.com (X.R.); simba695269042@163.com (Z.Z.); 2 Key Laboratory of Protection, Development and Utilization of Medicinal Resources in Liupanshan Area, Ministry of Education, School of Pharmacy, Ningxia Medical University, Yinchuan 750004, China; ssjyhhxx@163.com; 3 Department of Clinical Medicine, School of Clinical Medicine, Ningxia Medical University, Yinchuan 750004, China; 18161597728@163.com

**Keywords:** minute virus of canines, NP1, MCM7, protein interaction, viral replication

## Abstract

Minute virus of canines (MVC), which is a member of the Bocaparvovirus genus, is a non-enveloped, single-stranded DNA virus that causes respiratory and gastrointestinal disease in canines, as well as causing infertility and fetal death in pregnant dogs. The non-structural small protein NP1 of bocaparvoviruses is a unique feature that distinguishes the bocaparvovirus subfamily from other parvovirus subfamilies. In the life cycle of the MVC, NP1 plays an indispensable role in viral DNA replication and pre-mRNA processing. Currently, there is a paucity of studies reporting the characterization of host cell proteins interacting with NP1 during MVC replication. In this study, we screened and identified host cell proteins interacting with MVC-NP1 through immunoprecipitation (IP) combined with liquid chromatography and tandem mass spectrometry (LC-MS/MS) analysis; MCM7 (Mini-chromosome Maintenance Protein 7) has been identified and confirmed to interact directly with NP1 through its N-terminal domain. Furthermore, functional studies reveal that MCM7 is essential in MVC replication. The knockdown of MCM7 decreased the expression of this MVC protein significantly, as well as suppressing MVC replication by arresting the cell cycle in the G0/G1 phase during infection. Conversely, up-regulating MCM7 can rehabilitate the expression of MVC proteins, as well as supporting MVC replication. In conclusion, this study elucidates the interaction between the NP1 protein of MVC and the host factor MCM7, demonstrating that MCM7 is a key factor in the replication process of MVC. These findings provide a potential target for future antiviral therapy.

## 1. Introduction

Viruses are intracellular parasites that must encode a minimal number of proteins to complete their infectious life cycle and infect hosts. The International Committee on Taxonomy of Viruses classifies viruses in the Parvoviridae family that infect vertebrates as the Parvovirinae subfamily, which is divided into five genera—the Parvoviruses, Dependoviruses, Amdoviruses, Erythroviruses, and Bocaviruses [1,2]. Minute virus of canines (MVC) is a non-enveloped, single-stranded DNA virus and a member of the Bocaparvovirus genus [3]. MVC is also known as canine parvovirus type 1 (CPV-1) or canine minute virus/canine bocavirus (CnMV) [4,5]. MVC not only causes gastrointestinal and respiratory tract diseases in canines but also leads to infertility and fetal mortality in pregnant dogs; notably, young canines exhibit high susceptibility to this virus [6,7]. Moreover, MVC has also been linked to canine hepatitis [8,9].

MVC exhibits efficient replication in Walter Reed Dog (WRD) cells, yielding high-titer viral particles [10]. Building upon prior work, we reported the full-length infectious MVC clone pIMVC (GenBank: FJ214110), whose genome consists of 5404 nucleotides and has distinct palindromic hairpins at the left and right ends of the genome at 183 nt and 198 nt, respectively [3]. Like other parvoviruses, the genetic structure of MVC contains three open reading frames (ORFs). ORF1 in the left half of the genome encodes non-structural protein NS1, while ORF2 in the right half encodes the capsid proteins VP1 and VP2 [11]. A small ORF3 encodes a non-structural protein NP1, which is located between ORF1 and ORF2 and partially overlaps with the NS1 gene. Furthermore, NP1 localizes to the viral DNA replication centers in the nucleus and plays a crucial role in viral DNA replication [3]. NP1 is indispensable for viral DNA replication, and the BPV1 and HBoV1 NP1 proteins can replace MVC NP1 to support MVC DNA replication [3,12]. The mechanism defining how NP1 facilitates bocaparvovirus DNA replication remains largely unknown. It has been revealed that NP1 plays an important role in processing viral precursor mRNA (pre-mRNA) to mature viral mRNA polyadenylated at the distal polyadenylation site and is therefore important for capsid protein expression [13,14,15]. The NP1 proteins of both MVC and HBoV1 also facilitate the splicing of the 3D/3A intron that lies immediately upstream of the proximal polyadenylation (pA)p site; both of these processes are necessary to gain proper access to the capsid gene ORF [14,15]. Additionally, the C-terminal region of three of the MVC NS proteins is generated from mRNAs spliced at the third intron; thus, their expression is also facilitated by MVC NP1 [16].

Viruses recruit host proteins as part of their replication and spreading mechanisms. Many studies have attempted to elucidate virus–host interactions and their functional mechanisms. MVC is also heavily reliant on host cellular proteins during the processes of infection and replication. It has been reported that cleavage and polyadenylation specificity factor 6 (CPSF6) interacts with NP1 in transfected cells and cooperates with NP1 to play a crucial role in viral DNA replication and viral pre-mRNA processing [17]. HBoV1 NP1 also interacts with cellular RNA processing factor CPSF6 and regulates the polyadenylation of capsid protein-encoding mRNA, while the transport of HBoV1 NP1 to the nucleus is escorted by CPSF6 [18]. Moreover, a recent study revealed a novel mechanism by which HBoV1 NP1 enhances viral DNA replication through its direct interactions with Ku70 and RPA70 [19]. Notably, similar host factor-mediated replication mechanisms have been observed in other parvoviruses. For example, in adeno-associated virus (AAV), HBoV1 NP1 binds to host replication protein A (RPA) through a single-stranded DNA (ssDNA) binding domain, forming a heterologous complex that stabilizes viral replication intermediates and promotes rolling-circle replication by regulating the DNA binding kinetics of RPA. Experiments have shown that the loss of the ssDNA binding ability of NP1 or the mutation of the RPA binding site leads to a more than 70% decrease in viral DNA synthesis efficiency [20].

Investigating the role and regulatory mechanisms of host cell proteins in virus replication has emerged as a breakthrough in elucidating the mechanisms of virus replication and infection. However, it is currently unknown whether the NP1 protein of MVC interacts with host cell proteins and how host cell proteins should be used for virus replication. Given the important role of the NP1 protein in MVC replication, this study aimed to explore the interaction between viruses and host cells, using immunoprecipitation (IP) combined with liquid chromatography–tandem mass spectrometry (LC-MS/MS) analysis to identify host cell proteins that interact with the NP1 protein. Through co-immunoprecipitation (Co-IP) and GST-pulldown assays, we confirmed that the MCM7 protein could interact with NP1. In eukaryotes, MCM7 is a part of the origin recognition complex with other MCM proteins and is involved in working with cell division cycle proteins (CDCs) to arrest cells in the S phase and to activate the cell cycle checkpoint [21]. In this way, MCM7 regulates cell division from the S phase to the end of DNA replication, ensuring that only one replication occurs during a cell cycle, thereby maintaining the precision of the genetic material and the normal functioning of the cell [22]. In addition, MCM7 also participates in mRNA transcription and the regulation of DNA damage [23]. Therefore, the present study focuses on the interaction between host cell protein MCM7 and viral protein NP1, further exploring the significant role of MCM7 in MVC replication, which provides some theoretical support for a subsequent in-depth study of the replication mechanism of MVC.

## 2. Materials and Methods

### 2.1. Cells and Viruses

The primordial Walter Reed canine cell/3873D (WRD) cell line and minute virus of canines (MVC) were generously provided by Professor Jianming Qiu from the Department of Microbiology, University of Kansas Medical Center. COS-1 cells (monkey kidney epithelial cell line) were maintained in our laboratory. The cells were cultured in Dulbecco’s modified Eagle’s medium (DMEM; 11960044, Gibco, Grand Island, NY, USA) containing 10% fetal bovine serum (FBS; Cat NO. FS201-02, Trans Gen Biotech, Beijing, China) and 1% penicillin–streptomycin (Cat No.15140122, Thermo Fisher Scientific, Waltham, MA, USA) at 37 °C with 5% CO_2_. High-titer stock of MVC was produced in WRD cells using seed stock and was stored at −80 °C. For infection, WRD cells were infected with MVC for 1.5 h at an MOI of 1. After removing the virus solution, cells were washed three times with 1 × PBS and then incubated with fresh DMEM containing 10% FBS for the designated time.

### 2.2. Plasmids and Transfection

The plasmid expressing NP1 (pXJ40-Flag-NP1) was previously constructed and stored in our laboratory [24]. MCM7 and its four associated structural domains were cloned into pCMV-HA vectors, with empty vectors being used as controls. All expression vectors were constructed by Sangon Biotech Co., Ltd. (Shanghai, China) and were validated by double enzymatic digestion and sequencing. The primers used in this article are listed in Table 1. Polyethylenimine (PEI, Polysciences, Warrington, PA, USA) transfection reagent was employed for all plasmid transfections.

### 2.3. Immunoprecipitation (IP) and Liquid Chromatography–Tandem Mass Spectrometry (LC-MS/MS)

WRD cells were cultured in 100 mm dishes, infected with MVC for 48 h, then harvested and lysed on ice for 15 min in NP-40 lysis buffer (Cat No P0013F, Beyotime, Shanghai, China) containing protease inhibitors. The lysate was subsequently clarified by centrifugation at 14,000× *g* for 15 min at 4 °C. The supernatants were transferred to new tubes and incubated with anti-NP1 or rabbit IgG antibodies at 4 °C for 4 h, followed by the addition of Protein A/G PLUS668 agarose beads (Santa Cruz Biotech, sc-2003, Dallas, TX, USA), before being incubated overnight. The agarose bead–antigen–antibody complexes were washed five times with pre-cooled PBST buffer and boiled in a metal bath at 95 °C for 10 min. Subsequently, the immune complexes were separated by 10% sodium dodecyl sulfate-polyacrylamide gel electrophoresis (SDS-PAGE), stained with Coomassie brilliant blue for 2 h, and then destained. The gels were sent to Beijing Qinglian Baiao Biotechnology Co., Ltd. (Beijing, China) for full-lane MS analysis.

### 2.4. Western Blot Analysis

Cells were washed three times with pre-chilled 1 × PBS and lysed on ice for 30 min using lysis buffer (Cat No. KGP701, KeyGEN Biotech, San Francisco, CA, USA). After the lysates were clarified by centrifugation at 14,000× *g* for 15 min at 4 °C, protein quantification was performed using the bicinchoninic acid (BCA) assay. SDS-PAGE separated equivalent amounts of protein, which were then transferred to PVDF membranes (Cat No. 0000229835, Millipore, Burlington, MA, USA). The membranes were blocked with 5% non-fat milk and were subsequently incubated with the corresponding primary antibodies overnight at 4 °C, followed by incubation with horseradish peroxidase-conjugated secondary antibodies for 1 h at room temperature. Protein bands were visualized using Super ECL Prime (Cat No. SW181-01, SEVEN Biotech, Beijing China), and chemiluminescent signals were captured with the Azure 300 Imaging System (San Jose, CA, USA). Relative expression levels were quantified using ImageJ software v1.46 (Bethesda, MD, USA). The primary antibodies used for Western blotting are as follows: anti-MCM7 (Rabbit pAb, 11225-1-AP; WB: 1:10,000), anti-MCM7 (Mouse mAb, 67446-1-Ig; WB: 1:10,000), anti-MCM3 (Rabbit pAb, 84792-5-RR; WB: 1:10,000), anti-MCM5 (Rabbit pAb, 11703-1-AP; WB: 1:3000), anti-GAPDH (Rabit pAb; WB: 1:10,000), anti-HA (66006-1-Ig; WB: 1:1000; IP: 3 μg), and anti-Flag (66008-3-Ig; WB: 1:1000; IP: 3 μg) obtained from Proteintech (Wuhan, China); meanwhile, anti-NS1 (Rabbit pAb, 18929-1; WB: 1:1000), anti-VP2 (Rabbit pAb, 20351-1; WB: 1:1500), and anti-NP1 antibody (Mouse monoclonal, 1C19-1; 1:1500) were developed in collaboration with Abmart (Shanghai, China). The anti-GST antibody (AE007; WB: 1:3000) was obtained from ABclonal (Wuhan, China). The following secondary antibodies were used for Western blotting: Goat-anti-mouse (ZB-2305; WB: 1:5000) and Goat-anti-rabbit (ZB-2301; WB: 1:5000), both of which were obtained from ZSGB-BIO (Beijing, China).

### 2.5. Co-Immunoprecipitation (Co-IP) Assay

Co-transfected COS-1 cells or MVC-infected WRD cells were collected and lysed in NP-40 lysis buffer (Cat No. P0013F, Beyotime, Shanghai, China) supplemented with protease inhibitors, followed by ultrasonic disruption. The lysates were centrifuged at 12,000× *g* for 10 min at 4 °C, and supernatants were collected. A total of 10% of the supernatants was taken as the input control, mixed with 1 × loading buffer, and denatured at 95 °C for 10 min. For immunoprecipitation, cell lysates were separately probed with the anti-NP1, anti-MCM7, anti-MCM3, anti-MCM5, anti-Flag, and anti-HA antibodies at the best recommended ratio, or with normal species-matched IgG controls rotated overnight at 4 °C. Subsequently, an appropriate volume (100 μL) of protein A/G PLUS-Agarose beads (Cat No. PR40025, Proteintech, Wuhan, China) was added and incubated at 4 °C for 4 h in order to capture the antigen–antibody complex. The agarose bead–antigen–antibody complex was collected and washed 5–6 times with pre-cooled PBST buffer, before being immunoblotted using a Western blot.

### 2.6. GST-Pulldown

The GST pull-down experiment was conducted according to the manufacturer’s instructions. The recombinant MCM7 N-terminal domain fusion protein from bacterial lysates was incubated with 100 μL GST affinity resins (Cat No. DP201, TransGen, Illkirch-Graffenstaden, France), which had been pre-washed three times with PBS, under constant rotation at 4 °C overnight. After the overnight binding, the MCM7 N-terminal domain was successfully bound to the GST affinity resin, followed by five washes with pre-chilled PBS buffer. The affinity resin mixture was incubated with cell lysates from MVC-infected WRD cells at 4 °C for a duration of 4 h. Subsequently, the affinity resins were washed five times with PBS buffer and were eluted by boiling in 1× loading buffer (Cat No. CW 0027S, CoWin Biotech, Taizhou, China) at 95 °C for 10 min, followed by Western blot analysis to detect target proteins.

### 2.7. Quantitative Real-Time PCR (qRT-PCR)

For mRNA detection, total RNA was extracted from WRD cells using Trizol reagent (Invitrogen, 15596026) according to the manufacturer′s protocol. cDNA was reverse-transcribed from 1 μg of mRNA using a PrimeScript RT reagent Kit (Cat No. RR047Q, Takara, Kyoto, Japan). qPCR reactions (20 µL total volume) contained 2 µL of cDNA, 10 µL of SGExcel FastSYBR Mix (Cat No. 4444556, Thermo Fisher Scientific, Waltham, MA, USA), and 0.4 µM of each primer, and the reaction was performed on qPCR soft (Analytik Jena AG, Jena, Germany) to assess mRNA levels. The mRNA expression levels of the respective genes were expressed as a ratio compared to GAPDH in the same sample by calculating the cycle threshold (Ct) value in qRT-PCR amplification, of which 1.5 μL of diluted cDNA was used as a template.

For viral DNA detection, vDNA in infected WRD cells was extracted using a Quick-DNA™ viral kit (Cat No. D3015, ZYMO Research, Irvine, CA, USA), and the virus supernatant was collected. Viral DNA levels and extracellular viral copies were calculated using standard curves of PCR amplification obtained from serial dilutions of the pI-MVC plasmid [3]. The standard plasmid pI-MVC was quantified by spectrophotometric analysis (NanoDrop ND-1000, Thermo Fisher Scientific, Waltham, MA, USA) and converted to copy number. Ten-fold serial dilutions of the plasmids were used for absolute quantification. The calculation of viral copy number is described in detail in previous articles [24]. The primer sequences were as follows: MCM7 (F:5′-CCAGCCAGGAGTGCCAGAC-3′; R:5′-ACGGTGATGCTACGAGGGATG-3′); NP1 (F:5′-CTCTTCCTGCGTTCTGTG-3′; R:5′-GCCATCTACCTCCATTGC-3′); and GAPDH (F:5′-GCTGAGTATGTTGTGGAGT-3′; R:5′-GCAGAAGGAGCAGAGATG-3′).

### 2.8. Short Hairpin (sh) RNA-Mediated Gene Silencing

The short-hairpin RNA (shRNA) targeting the Canis lupus familiaris MCM7 gene (XM038668108) was designed and purchased from Genomeditech (Shanghai, China); the scrambled shRNA was used as a negative control. Negative-control lentivirus was used as an expression control to generate a non-silencing lentiviral stock to optimize expression conditions. ShRNAs specific to the MCM7 gene used in the study are shown in Table 2. They were inserted into the lentiviral PGMLV-hU6-MCS-CMV-puro vector, containing a puromycin-resistant gene. For lentiviral packaging, the constructed lentiviral vector and its helper packaging from the original vector plasmid were co-transfected into 293T cells using HG transgene reagent; the enhancing buffer was added after 10~12 h of transfection, followed by the replacement of the fresh medium after 8 h. The cells were further incubated for 48 h. The cellular supernatant enriched with lentiviral particles was collected and then concentrated to obtain high-titer lentiviral concentrates, and the viral titers were measured and calibrated in the 293T cells. The final titer of the recombinant virus was 5 × 10^8^ transducing units (TU/mL), and the experimental recombinant lentivirus was stored at −80 °C. Stable knockdown cell lines were generated by lentiviral infection followed by puromycin selection. WRD cells were infected with lentivirus-containing supernatant for 24 h in the presence of 1 μg/mL polybrene (Cat No. H8761, Solarbio, Beijing, China). Cells were selected with puromycin at 1 μg/mL during cell passage.

### 2.9. Cell Cycle Detection by Flow Cytometry

The cell cycle of WRD cells was evaluated by flow cytometry. Cells were seeded on 60 mm dishes and incubated at 37 °C in 5% CO_2_ at constant temperature for 24 and 48 h after being infected with MVC. The following cell treatment was performed according to the instructions of the cell cycle kit (Cat No. CCS012, Liank Bio, Hangzhou, China). The supernatant was aspirated and washed with PBS, digested with trypsin, and centrifuged at 1000 rpm for 5 min to obtain the cell precipitate. It was washed again with PBS, before being centrifuged again. The cell suspension was then fixed with 75% anhydrous ethanol and stored overnight at −20 °C. On the day of detection, the fixed cells were centrifuged to remove the ethanol, and the cells were dislodged by tapping the wall of the tube and being incubated with 3 mL of PBS at room temperature for 15 min to rehydrate the cells. After centrifugation, the supernatant was removed, and 1 mL of DNA staining solution was added and mixed well, before being incubated in the dark at room temperature for 30 min. The lowest loading rate was then selected for cell cycle detection by flow cytometry (Beckman, Palo Alto, CA, USA).

Gating strategy: First, the main cell population is gated on the forward scatter area (FSC-A) vs. side scatter area (SSC-A) scatter plot to exclude cell clumps and aggregates. Second, doublets are excluded using the FSC-A vs. forward scatter height (FSC-H) scatter plot. Single cells exhibit a linear relationship between FSC-A and FSC-H. In contrast, doublets (two adherent cells) have a DNA content of 4n, with an increased volume but unchanged peak height, which causes them to deviate from the diagonal line. Therefore, the diagonal region is gated to exclude doublets. Finally, propidium iodide area (PI-A) histogram analysis is performed to distinguish G0/G1-, S-, and G2/M-phase cells, followed by cell cycle fitting.

### 2.10. Statistical Analysis

All data were analyzed by Prism (GraphPad Prism 5, San Diego, CA, USA) software through one-way ANOVA and unpaired t-test. All functional experiments were performed at least 3 times independently, and representative data are shown. Data represent the mean ± standard error of three independent experiments (SEM). *p* < 0.05 was considered statistically significant (* *p* < 0.05, ** *p* <0.01, *** *p* < 0.001; **** *p* < 0.0001; ns: no significant). Figures were generated using Adobe Photoshop CC v2014 (San Jose, CA, USA).

## 3. Results

### 3.1. Screening and Identification of Host Cell Proteins Interacting with the MVC NP1 Protein

**Figure 1:**
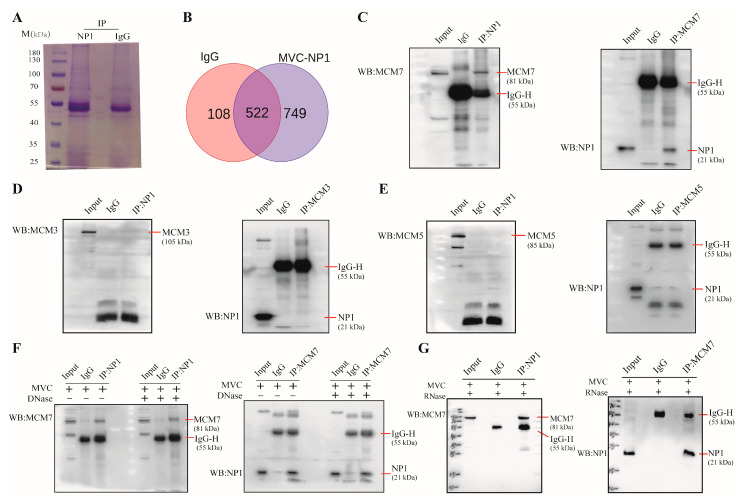
Screening and identification of host cell proteins that interact with MVC NP1. (**A**) WRD cells were infected with MVC at an MOI of 1 for 48 h and lysed on ice using NP40 lysis buffer. Cell lysates were first incubated with a negative control anti-IgG antibody, followed by incubation of the purified lysates with an anti-NP1 antibody. Immunoprecipitated proteins were separated by 10% SDS-PAGE, stained with Coomassie Brilliant Blue, and then subjected to liquid chromatography and tandem mass spectrometry (LC-MS/MS) analysis. (**B**) Venn diagram of host proteins bound to MVC NP1. (**C**–**G**) Identification of cellular proteins interacting with MVC NP1 via Co-IP assays. (**C**) WRD cells were infected with MVC (MOI = 1) for 48 h, lysed, and subjected to immunoprecipitation using anti-NP1 or anti-MCM7 antibodies, respectively, as described. Immunoblots were probed with anti-MCM7 and anti-NP1 antibodies. Total cell lysates were used as an input control, and IgG served as a negative control. (**D**,**E**) Similarly to (**C**), Co-IP assays were conducted using anti-NP1 or anti-MCM3 antibody (**D**) and anti-NP1 or anti-MCM5 antibody (**E**), and detection was performed by Western blot using anti-MCM3, anti-MCM5, or anti-NP1 antibodies. (**F**) WRD cells infected with MVC (MOI = 1) were lysed and subjected to treatment with or without DNase. Subsequently, immunoprecipitation was performed using anti-MCM7 or anti-NP1 antibodies, followed by immunoblotting with anti-NP1 or anti-MCM7 antibodies, respectively. IgG was used as a negative control. (**G**) Similarly to (**F**), WRD cells infected with MVC at an MOI of 1 were lysed and treated with RNase (used at a final concentration of 5 μg/mL). Then, immunoprecipitation was carried out using anti-MCM7 or anti-NP1 antibodies, followed by immunoblotting with anti-NP1 or anti-MCM7 antibodies, respectively. IgG was used as a negative control. The experiment was independently repeated three times, and the representative images are displayed.

As previously reported, MVC-NP1 plays a critical role in MVC DNA replication [3] and regulates viral mRNA processing [13,14]. To further investigate the function of NP1 in viral DNA replication, immunoprecipitation (IP) was performed along with liquid chromatography and tandem mass spectrometry (LC-MS/MS) analysis to identify cellular proteins that interact with NP1 during MVC DNA replication (Figure 1). The lysates of WRD cells infected with MVC were immunoprecipitated (IP) using an anti-NP1 antibody and analyzed using sodium dodecyl-sulfate polyacrylamide gel electrophoresis (SDS-PAGE) combined with Coomassie blue staining (Figure 1A). The gel was sent to Beijing Qinglian Baiao Biotechnology Co., Ltd. for LC-MS/MS analysis. The proteomics analysis results showed that a total of 522 host cell candidate proteins were specifically bound to MVC NP1 in WRD cells infected with MVC (Figure 1B). The NP1 interacting proteins were categorized by comparison to negative control IgG, and Gene Ontology (GO) term enrichment analysis revealed that 10 proteins were involved in DNA replication. We selected MCM3, MCM5, and MCM7 from the Mini-chromosome Maintenance (MCM) protein family for reciprocal Co-IP validation to confirm their interactions with NP1. The results showed that anti-NP1-conjugated beads could pull down MCM7 in the absence of DNA or RNA mediation (Figure 1C,F,G), whereas MCM3 and MCM5 did not interact with NP1 (Figure 1D,E).

### 3.2. MCM7 Interacts with NP1 Through Its N-Terminal Domain

**Figure 2:**
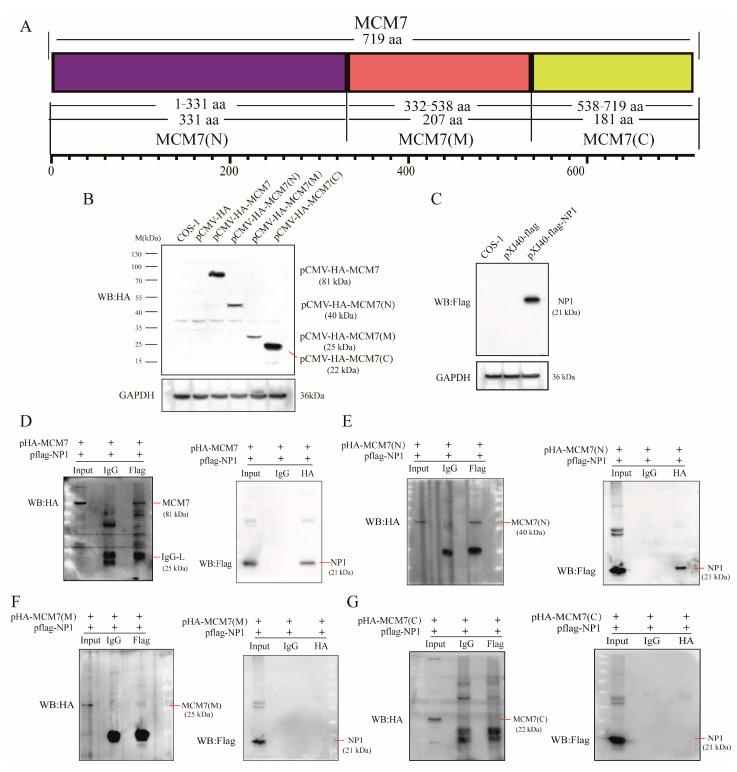
The interaction between NP1 and MCM7 is mediated by the N-terminal domain. (**A**) Schematic diagram of MCM7 domains. The N-terminal domain (MCM7-N), the Middle domain (MCM7-M), and the C-terminal domain (MCM7-C) of MCM7 are diagrammed and distinguished by different colors. (**B**,**C**) Validation of transfection efficiency of eukaryotic recombinant vectors in COS-1 cells via Western blot. COS-1 cells were transfected with pCMV-HA-MCM7, pCMV-HA-MCM7(N), pCMV-HA-MCM7(M), pCMV-HA-MCM7(C), and pCMV-HA (as a vector control), or pXJ40-Flag-NP1 and pXJ40-Flag (as vector control), respectively; cell lysates were analyzed at 48 h post-transfection. (**D**–**G**) Identification of the interaction between NP1 and MCM7 by Co-IP assay in COS-1 cells. (**D**) Eukaryotic plasmids pCMV-HA-MCM7(pHA-MCM7) and pXJ40-Flag-NP1(pFlag-NP1) were co-transfected into COS-1 cells as indicated. Reciprocal Co-IP experiments were performed using rabbit anti-HA or mouse anti-Flag antibodies to detect interactions between MCM7 and NP1. Mouse or rabbit IgG serves as a negative control. (**E**–**G**) Verification of the specific domains of MCM7 that interact with NP1 by Co-IP assay. The pCMV-HA-MCM7(N), pCMV-HA-MCM7(M), or pCMV-HA-MCM7(C) plasmids were co-transfected with the pXJ40-Flag-NP1 plasmid into COS-1 cells, respectively. Subsequent treatments were the same as those described in (**D**), followed by a reciprocal Co-IP assay to verify their interaction. All the experiments were independently repeated three times, and the representative images are shown.

Host cellular DNA replication factors are always associated with viral replication centers. As it was previously reported that MCM2 plays an essential role in viral DNA replication, as a component of the Mini-chromosome Maintenance (MCM) complex, it is recruited to the viral DNA replication origin by the viral DNA replication origin (Ori)-binding protein [25,26]. MCM7 is a crucial component of MCM complexes, which are essential for chromosomal DNA replication [27,28]. MCM7 contains three structural domains (Figure 2A); the N-terminal region is responsible for DNA binding and protein multimerization, while the C-terminal region is a predicted helix–turn–helix motif with DNA helicase activity [28,29,30]. To further confirm the authentic interaction between them and to identify the specific domain of MCM7 involved in binding to NP1, we first cloned full-length MCM7 and its three domain mutants into pCMV-HA eukaryotic expression vectors, with validation results being shown in (Figure 2B). Concurrently, NP1 was cloned into the pXJ40-Flag eukaryotic expression vector (Figure 2C). The HA-tagged MCM7 plasmid was co-transfected with pFlag-NP1 into COS-1 cells for verification. Two days post-transfection, cell lysates were subjected to Co-IP assays using specific antibodies and Protein A/G agarose beads. Not unexpectedly, the Co-IP results (Figure 2D) showed an interaction between MCM7 and NP1, mirroring the results observed in MVC-infected WRD cells, thus confirming the specific interaction between MVC NP1 and MCM7. To map the NP1-binding region within MCM7, we constructed three HA-tagged MCM7 truncated mutants, designated as pCMV-HA-MCM7(N), pCMV-HA-MCM7(M), and pCMV-HA-MCM7(C), respectively. These mutants were co-transfected with pXJ40-Flag-NP1 into COS-1 cells for Co-IP assays. Reciprocal Co-IP experiments revealed that only the N-terminal domain of MCM7 was sufficient for binding to the viral NP1 (Figure 2E), whereas the middle domain (Figure 2F) and C-terminal domain (Figure 2G) of MCM7 both lacked binding capacity. These findings indicate that the N-terminal domain of MCM7 is critical for its interaction with the viral NP1 protein.

**Figure 3:**
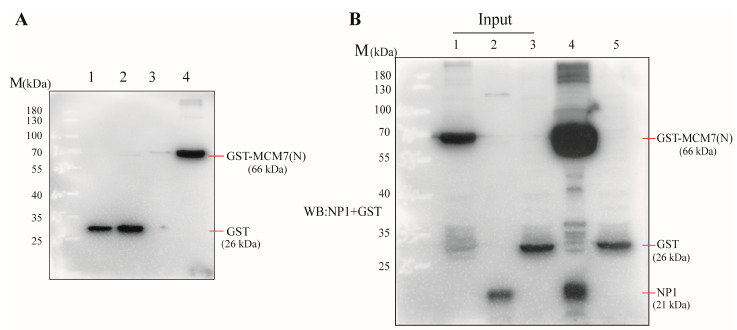
Verification of the interaction between MCM7(N) and NP1 in vitro. (**A**) Induction of GST-MCM7(N) fusion protein analyzed by Western blot. M: protein maker; Lane 1: pGEX-4T-1 empty vector before induction; Lane 2: pGEX-4T-1 empty vector after induction; Lane 3: pGEX-4T-1-MCM7(N) recombinant vector before induction; Lane 4: pGEX-4T-1-MCM7(N) recombinant vector after induction. (**B**) GST-pulldown assay. GST and GST-MCM7(N) were separately expressed in *E. coli* and immobilized on glutathione Sepharose beads. The immobilized GST-tagged proteins were then incubated with NP1 protein lysates from MVC-infected WRD cells. Pull-down proteins were analyzed by Western blot using anti-GST and anti-NP1 antibodies. M: protein maker; Lane 1: pGEX-4T-1-MCM7(N) recombinant vector induction; Lane 2: MVC-infected WRD cell lysate; Lane 3: pGEX-4T-1 empty vector induction; Lane 4: co-incubation of pGEX-4T-1-MCM7(N) fusion proteins with lysate from MVC-infected WRD cells; Lane 5: co-incubation of protein from pGEX-4T-1 empty vector with lysates from MVC-infected WRD cells. All the experiments were independently repeated three times, and the representative images are displayed.

Previous studies have elucidated the role of the N-terminal portion in MCM multimerization, ssDNA and dsDNA binding, and ATPase activity [28,31]. Building upon the functional significance of the MCM7 N-terminal domain and the aforementioned Co-IP results, we sought to further validate the interaction between MCM7(N) and NP1 using a prokaryotic expression system. The recombinant GST-MCM7(N) fusion protein was successfully induced in *Escherichia coli* under optimized conditions (0.8 mM IPTG at 26 °C). The results are shown in (Figure 3A). The GST-pulldown assay confirmed a physical interaction between the MCM7 N-terminus and NP1 (Figure 3B). Taken together, these results provide biochemical evidence that the N-terminal of MCM7 binds to the MVC NP1 protein.

### 3.3. Knockdown of MCM7 Suppresses Viral Protein Expression and MVC Replication

**Figure 4:**
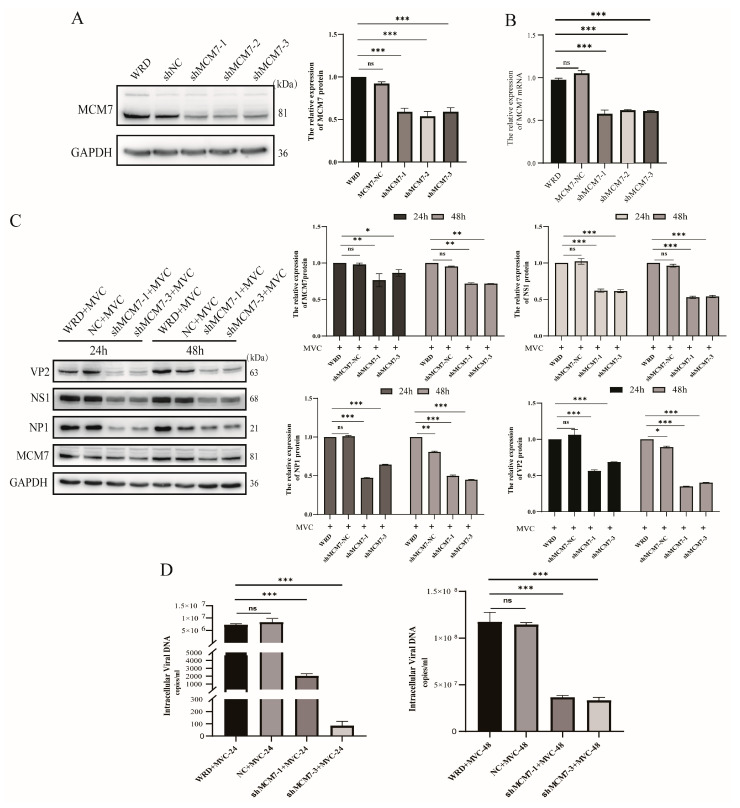
Effect of MCM7 knockdown on MVC replication. (**A**,**B**). Confirmation of MCM7 knockdown in WRD cells using Western blot and qPCR assays, respectively. WRD cells were infected with shRNA-mediated lentivirus, continuously screened with puromycin at 1 μg/mL for 3 passages, and then harvested to extract proteins and mRNA for detection. (**C**). The impact of MCM7 knockdown on MVC replication in MVC-infected cells. The shMCM7-1/-3 cells were infected with MVC (MOI = 1) for 24 and 48 h, and NS1, NP1, and VP2 protein expressions were analyzed via Western blot. (**D**). The intracellular vDNA was extracted at 24 and 48 h post-infection and subjected to qRT-PCR. All data were normalized to GAPDH, which was used as an internal control. Statistical analysis was performed using one-way ANOVA, with comparisons made against the negative control group (MVC-infected WRD). Protein levels were quantified using ImageJ software v1.46 (Bethesda, MD, USA), and the statistical results are presented on the right side of these figures. Error bars represent the SD of the means from three independent experiments (ns: no statistical significance; * *p* < 0.05, ** *p* < 0.01, *** *p* < 0.001).

Previously, we have comprehensively validated the interaction between the NP1 and MCM7 proteins. To elucidate the involvement of MCM7 in MVC virus replication, a loss-of-function analysis was conducted to explore the biological role of MCM7 in MVC replication. To interfere with the expression of the MCM7 protein, a screening process was initiated in which lentiviral particles were packed with shMCM7 (short hairpin RNA targeting MCM7) and the negative control shNC (non-targeting short hairpin RNA) in WRD cell lines. As shown in (Figure 4A,B), compared with the normal WRD cells and the NC group, both protein and mRNA expression levels in the MCM7-interfered group were significantly decreased. In subsequent experiments, two of the stable silencing MCM7 experimental groups were selected—shMCM7-1 and shMCM7-3 stable cell lines. Interestingly, silencing MCM7 exerted a significant inhibitory effect on MVC replication, as evidenced by the significant decrease in the expression of both the structural (VP2) and non-structural proteins (NS1 and NP1) of MVC compared to shNC-treated and WRD cells (Figure 4C). In addition, we extracted the intracellular viral nucleic acids using a viral nucleic acid extraction kit and detected their expression via a quantitative polymerase chain reaction (qPCR) assay. Similarly, the qPCR results displayed in Figure 4D also indicate a significant reduction (*p* < 0.05) in the copy number of viral nucleic acid in the MCM7-silenced cell lines at 24 and 48 h post-MVC infection. Obviously, knocking down MCM7 led to a substantial decrease in both the detectable protein and viral nucleic acid levels of MVC. Therefore, we further investigated the effect of MCM7 overexpression on viral replication.

### 3.4. Overexpression of MCM7 Promotes Viral Protein Expression and MVC Replication

**Figure 5:**
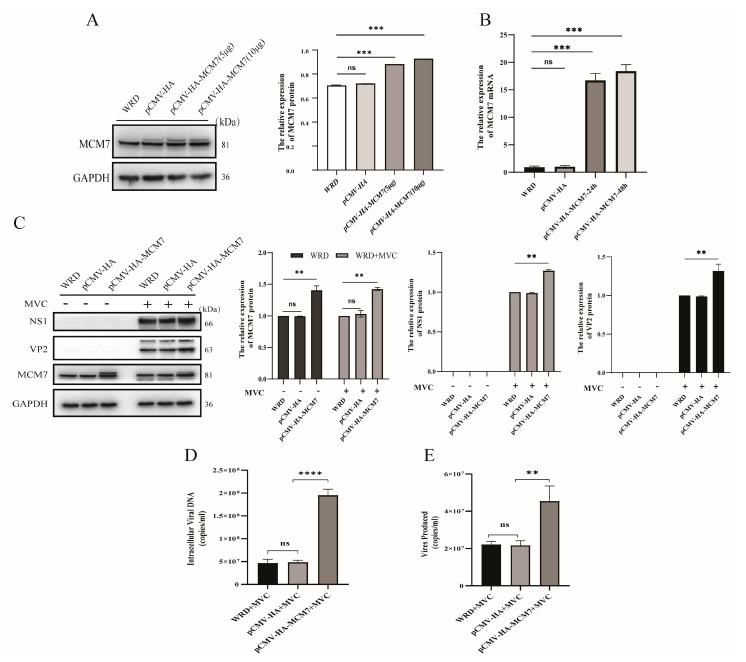
Effect of MCM7 overexpression on MVC virus replication. (**A**,**B**). Confirmation of MCM7 overexpression in WRD cells. WRD cells were transfected with 5 μg and 10 μg of pCMV-HA (negative control) and pHA-MCM7 plasmid, respectively, for 24 h. Subsequently, the expression levels of MCM7 protein and mRNA were assessed using Western blot and qPCR. (**C**). The effects of the overexpression of MCM7 on MVC replication in MVC-infected cells. WRD cells were transfected with pHA-MCM7 (5 μg) for 24 h and then infected with MVC (MOI = 1) for another 24 h. The levels of viral NS1 and VP2 proteins were detected by Western blot. (**D**,**E**). Effects of the overexpression of MCM7 in infected cells on the production of MVC. At 24 h post-MVC infection, qPCR was employed to detect the copy number of virions released into the culture supernatants and the content of viral nucleic acids in normal WRD cells and MCM7-overexpressing cells. All data were normalized to GAPDH, which was used as an internal control. Statistical analysis was performed using one-way ANOVA, with comparisons made against the negative control group (MVC-infected WRD). Protein levels were quantified using ImageJ, software v1.46 (Bethesda, MD, USA), and the statistical results are presented on the right side of these figures. Error bars represent the SD of the means from three independent experiments (ns: no statistical significance; ** *p* < 0.01, *** *p* < 0.001, **** *p* < 0.0001).

The above results demonstrated that inhibition of MCM7 significantly suppressed MVC replication, as evidenced by reduced viral protein expression and decreased virion copy number. To investigate whether MCM7 overexpression would potentiate MVC replication, we first transfected the recombinant overexpression plasmid pCMV-HA-MCM7 into WRD cells. Following a 24 h incubation, we assessed the expression of MCM7. The results (Figure 5A,B) showed that compared with the control group of WRD cells, the protein and mRNA expression levels of MCM7 in WRD cells transfected with 5 μg or 10 μg of MCM7 overexpression plasmid were significantly increased (*p* < 0.05). Based on the clarification that the overexpression plasmid of MCM7 was successfully expressed in WRD cells, WRD cells were transfected with the pCMV-HA-MCM7 plasmid for 24 h before MVC viral infection. Subsequently, the viral proteins and viral DNA (vDNA) extracted from infected cells were assessed by Western blot and qRT-PCR, respectively. As expected, the overexpression of pHA-MCM7 (5 μg) showed comparable increases in both NS1 and VP2 proteins compared with the pHA-vector group (control) (Figure 5C). Meanwhile, the effect of the overexpression of MCM7 on viral production is also shown in (Figure 5D,E). We observed that compared with the pHA-vector control group, virion production increased by at least onefold in the MCM7 overexpression group. Generally speaking, these results indicate that the overexpression of MCM7 in WRD cells has positive effects on MVC replication.

**Figure 6:**
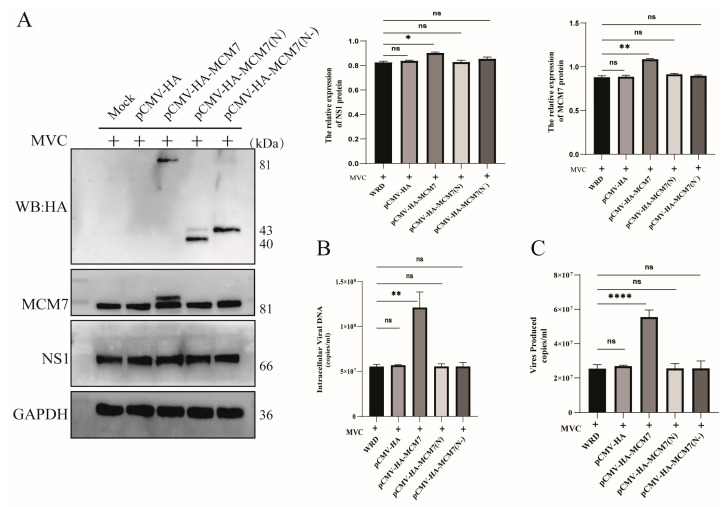
Effect of the interaction between MCM7 and NP1 on viral replication. (**A**) Western blotting (WB) was used to verify whether the plasmids encoding MCM7, the N-terminal domain of MCM7, and the N-terminal deletion mutant of MCM7 were successfully transfected, as well as to detect the expression of viral proteins and the expression of MCM7 itself. WRD cells were transfected with pCMV-HA, pHA-MCM7, pHA-MCM7(N), and pHA-MCM7(N-) plasmids, respectively. After 24 h of culture, the cells were infected with MVC and incubated for an additional 36 h. (**B**,**C**) qPCR was employed to detect the copy number of virions released into the culture supernatants and the content of viral nucleic acids in normal WRD cells and WRD cells transfected with each of the four plasmids, respectively. Statistical analysis was performed using one-way ANOVA, with comparisons made against the negative control group (MVC-infected WRD). Protein levels were quantified using ImageJ software v1.46 (Bethesda, MD, USA), and the statistical results are presented on the upper right corner of these figures. Error bars represent the SD of the means from three independent experiments (ns: no statistical significance; * *p* < 0.05, ** *p* < 0.01, **** *p* < 0.0001).

The aforementioned research has fully confirmed that the viral protein NP1 interacts with the host cellular protein MCM7; additionally, regulating the expression of MCM7 perturbs the expression of viral proteins as well as viral DNA replication. Thus, we venture to hypothesize that NP1 hijacks MCM7 to the viral replication center by binding to its N-terminal domain, thereby promoting viral DNA replication and viral protein expression. As shown in (Figure 6), the results demonstrated that compared with the transfection of the full-length MCM7 overexpression plasmid (pCMV-HA-MCM7), the transfection of either the plasmid encoding the N-terminal domain of MCM7 ((pCMV-HA-MCM7(N)) alone or the plasmid encoding the N-terminal deletion mutant of MCM7 ((pCMV-HA-MCM7(N-)) did not affect viral protein expression or viral replication. Meanwhile, we also detected changes in the expression of the host cellular protein MCM7. The results showed that the transfection of the plasmid encoding the N-terminal domain of MCM7 and the plasmid encoding the N-terminal deletion mutant of MCM7 did not affect the expression of MCM7 itself. This indicates that MCM7 exerts a regulatory effect on the expression of MVC viral proteins and viral replication in its intact form; this mechanism warrants further investigation.

**Figure 7:**
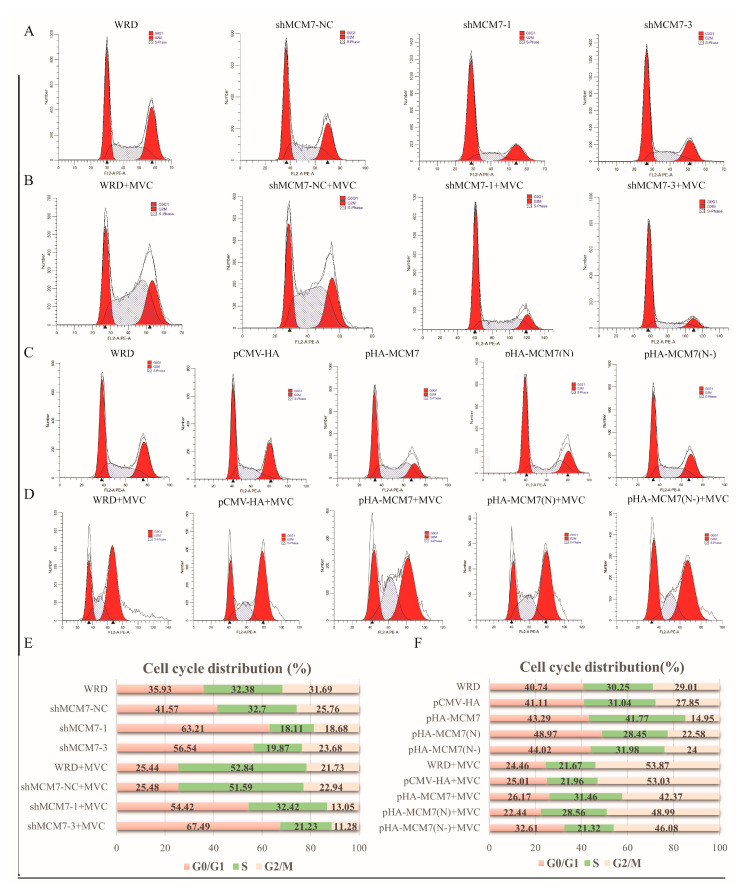
Host cell protein MCM7 regulates the cell cycle. (**A**–**D**) Cell cycle distribution profiles of WRD cells, either uninfected or infected with MVC. (**A**,**B**) WRD cells and MCM7-knockdown cells, either infected or uninfected with MVC, were collected after 24 h of culture, stained with propidium iodide (PI), and analyzed by flow cytometry. (**C**,**D**) WRD cells were transfected with pCMV-HA, pHA-MCM7, pHA-MCM7(N), and pHA-MCM7(N-) plasmids, respectively. After 24 h of culture, the cells were infected with MVC and incubated for an additional 36 h. Cells were then harvested, stained with propidium iodide (PI), and analyzed by flow cytometry. (**E**,**F**) The percentage of cells in each phase of the cell cycle is shown.

The results presented above suggested that the silencing of MCM7 inhibits the expression of MVC proteins and the replication of MVC, whereas the overexpression of MCM7 rescues these processes. Since the NP1 protein is required for viral DNA replication, MVC infection creates a prolonged S-phase environment for its own DNA replication [32]. We further reasoned that MCM7 may regulate viral protein expression and replication by interfering with the host cell cycle. As depicted in (Figure 7A,B,E), the silencing of the MCM7 gene leads to cell cycle arrest at the G0/G1 phase, accompanied by a significant decrease in the number of S-phase cells. Meanwhile, after transfecting WRD cells with the MCM7 overexpression plasmid, the number of S-phase cells increased by approximately 10% compared with the negative control group. However, the transfection of WRD cells with plasmids encoding only the N-terminal domain of MCM7 or the N-terminal domain deletion mutant of MCM7 alone did not cause significant changes in the cell cycle (Figure 7C,D,F). For parvoviruses, the S phase is a crucial period for them to hijack the cellular replication machinery [33]. Previous studies have reported that in the early stage of MVC infection in WRD cells, the proportion of cells in the S phase is the highest; however, as the infection time increases, the cell cycle gradually transitions from accumulation in the S phase to arrest in the G2/M phase [32,34], which is consistent with our research findings. Therefore, MCM7 functions, as a whole, to perturb the host cell cycle and thereby regulate the expression of MVC viral proteins, rather than mediating its regulatory effect on the virus through the interaction between the N domain of MCM7 and the viral NP1 protein.

## 4. Discussion

We have now confirmed that MCM7 is directly involved in MVC DNA replication. Notably, the MVC non-structural protein NP1 specifically interacts with MCM7, and MCM7 plays a central role in eukaryotic DNA replication [29]. Furthermore, functional studies reveal that MCM7 is essential in MVC replication. The knockdown of MCM7 decreased the expression of the MVC protein significantly and suppressed MVC replication by arresting the cell cycle in the G0/G1 phase during infection. Conversely, up-regulating MCM7 can rehabilitate the expression of the MVC protein, as well as support MVC replication. These findings provide a potential target for future antiviral therapy.

Here, our study reveals that the host cell protein MCM7 interacts with the non-structural protein NP1 of MVC. A thorough understanding of the physical interactions that occur between viral proteins and cellular proteins within an infected cell is necessary to elucidate the molecular mechanisms underlying viral replication. Previous studies have confirmed that the HBoV1 NP1 protein exerts its functions by interacting with host proteins and aids in the replication of the viral genome in host cells [17,19]. Therefore, it is of great significance to clarify the MVC replication mechanism by screening host cell proteins that interact with the NP1 protein. In this study, we first identified that MCM7 directly interacts with NP1 without the involvement of DNA or RNA in MVC-infected WRD cells, whereas MCM3 and MCM5 do not bind to NP1 (Figure 1C–G). Furthermore, as shown in (Figure 2), we co-transfected the exogenously expressed viral protein NP1 and host cell protein MCM7 into COS-1 cells and further confirmed the existence of sufficient interaction between the two via Co-IP. A canonical MCM protein sequence can be divided into three domains, including an N-terminal domain, an AAA+ domain, and a C-terminal domain. Among these domains, the N-terminal domain exhibits a marked binding preference for single-stranded DNA (ssDNA); meanwhile, eukaryotic MCM7 has a full length of 719 amino acids, is highly homologous to MCM proteins from terrestrial organisms, and its three structural domains are functionally equivalent [28,30,35]. To determine the specific structural domains of the host cell protein that bind to NP1, we generated the eukaryotic mutants of MCM7 (Figure 2A,B). Plasmids encoding the N-terminal domain of MCM7, which is the chromatin-binding region of MCM7; the central domain of MCM7, which is the central structural domain of MCM7 and the possible DNA deconjugation region [35,36]; and the C-terminal domain were, respectively, co-transfected with the NP1 eukaryotic expression vector into COS-1 cells. Co-IP results showed that the N-terminal domain of MCM7 interacted with the NP1 protein in an obvious way (Figure 2E–G). Finally, we further cloned the N-terminal domain of MCM7 into the prokaryotic expression vector pGEX-4T-1, confirming the robust binding between NP1 and the N-terminus of MCM7 via the GST-pulldown assay (Figure 3). Mini-chromosome Maintenance (MCM) proteins serve as key components of the pre-replication complex to initiate DNA replication in eukaryotes, which also play an important role in the regulation of viral DNA replication and are usually recruited to the origin of viral DNA replication after the viral invasion of the target cell, thus directly promoting viral replication, or interfering with the normal function of the MCMs and hindering the DNA replication of the host cell, thus indirectly promoting viral replication [37,38]. Thus, the MCM complex acts as an essential host replication factor involved in viral genome replication within host cells, such as the Hepatitis B virus (HBV) [39]. It has been reported that KSHV ORF59 modulates cellular DNA replication licensing through binding with MCM proteins to sabotage host DNA replication and give direct advantage to viral DNA replication [40]. In addition, Zhang et al. reported that pUL117, as a novel viral factor of Human cytomegalovirus (HCMV), can inhibit replication licensing by targeting the MCM helicase and block cellular DNA synthesis upon HCMV infection [41]. Therefore, as a component of the MCM2-7 hexamer (MCMs), MCM7 plays a central role in eukaryotic DNA replication [37,42]. Based on this, we hypothesize that MCM7, which interacts directly with NP1, may play an important role in the replication process of MVC; this mechanism warrants further investigation.

Interestingly, our study found that MCM7 can facilitate the expression of MVC viral proteins and DNA replication. The results demonstrate that in the absence of exogenous cellular stress, the downregulation of MCM7 protein levels had no significant impact on cell viability. This observation aligns with previous findings indicating that as long as a number of MCM proteins are available to occupy the replication origins, cell survival is not affected [43]. Furthermore, this result is consistent with the well-established conclusion that human cells may exhibit an at least a 10-fold excess of chromatin-bound MCM complexes and may proliferate normally as long as the replication of the normal origins is conserved [44,45]. Further studies have demonstrated that the silencing of MCM7 led to a significant reduction in both virion production and protein expression (Figure 4). Conversely, the overexpression of MCM7 can rehabilitate the expression of MVC proteins, which, in turn, promotes MVC replication (Figure 5). MCM7 interacts with other MCM proteins, including MCM2-6, to form a hexameric complex and, as a replicative DNA helicase, the MCM complex participates in the assembly of the pre-replication complex (pre–RC) and plays a critical role in the initiation and elongation of DNA replication [46,47]. Then, whether the regulation of viral protein expression and DNA replication by MCM7 is achieved through NP1 hijacking MCM7 to viral replication centers via binding to the N-terminal domain of MCM7. To address this, we conducted further studies and found that the regulation of viral DNA replication by MCM7 is not mediated through its interaction with NP1 (Figure 6). A previous report indicated that MVC-induced DDR supports the replication of the viral DNA by first arresting cell cycle progression at the S phase and subsequently impairing the cell cycle at the G2/M phase to prevent mitosis, which would lead to apoptotic cell death [48]. Indeed, by detecting the cell cycle of the relevant WRD cells, we have clarified that the knockdown of MCM7 arrests approximately 60% of WRD cells in the G1 phase, and the reduction in S-phase cells was detrimental to MVC DNA replication, while the overexpression of MCM7 increases the number of S-phase cells by roughly 10% (Figure 7). Ibarra et al. demonstrated that knocking down any one of the MCM complex subunits (MCM2-7) will lead to the dysfunction of the whole complex and reduce the backup capacity of DNA licensing, which then leads to the abnormal replication of DNA during the S phase and activates the DNA damage response (DDR) to stop the cell cycle [45]. For MVC, the S phase is a crucial period to carry out its own DNA replication; thus, MCM7 promotes the expression of MVC proteins and viral DNA replication by regulating the cell cycle.

In this study, we have fully demonstrated that the viral protein NP1 interacts with the host cellular protein MCM7, and that MCM7 facilitates the expression of viral proteins and viral replication by regulating the host cell cycle. However, the specific mechanism by which MCM7 regulates the host cell cycle to promote viral replication remains unclear, which requires further in-depth investigation in subsequent studies.

## 5. Conclusions

In summary, we identified a critical host factor, MCM7, that interacts with the MVC non-structural protein NP1. We also demonstrated that MCM7 plays a promotive role in the viral replication process. The findings of this study provide new insights into the role of host cellular factors in MVC replication and suggest that MCM7 may serve as a novel antiviral target.

## Figures and Tables

**Table 1:** Primer sequences are used to construct plasmids.

Plasmid	Primer Sequences (5′-3′)
pHA-MCM7	F: 5′-GCGAATTCGCGCCCGTCACGTGGG-3′
	R:5′-ATGCGGCCGCTTTGAATAGAATATAGCAATCTACAAACACTTTATTAGC-3′
pHA-MCM7(N)	F:5′-GCGAATTCATGGCCGTGAAGGACTACGT-3′
	R:5′-ATGCGGCCGCGTCCTCCTCTGTGATCTGTCTCAG-3′
pHA-MCM7(M)	F:5′-GCGAATTCTGTAATGTGCTGGGCCAGTCTC-3′
	R:5′-ATGCGGCCGCTTCTACGAGAAGCTGGCCGC-3′
pHA-MCM7(C)	F:5′-GCGAATTCTACGTGCACCAGCATTCTAGACAG-3′
	R:5′-ATGCGGCCGCCACGAAGGTGATCCTGGTCCG-3′
pHA-MCM7(N-)	F:5′-ACGCGTCGACGTCGACCTTTTACGAAAAGCTG-3′
	R:5′-CCGCTCGAGCTCGAGTCACACACGAAGGTGAT-3′

F: forward; R: reverse. The underlined letters indicate the restriction enzyme cleavage sites of *EcoR*I/*Not*I and *Sal*I/*Xhol*I for cloning.

**Table 2:** Targeting sequence of shMCM7.

No.	Target Sequence
NC	5′-TTCTCCGAACGTGTCACGT-3′
1	5′-GGAGATCTATGGACACGAAGA-3′
2	5′-GCAGACACGTGGTTCCAAATT-3′
3	5′-GGTCTCCTCTCTGAAACTTAC-3′

## Data Availability

The original contributions presented in this study are included in the article. Further inquiries can be directed to the corresponding author.
